# A 5-year follow-up of Achilles tendon reconstruction using a full-thickness graft processed with the clearant method and retrograde fixation in the calcaneus: a case study of an amateur soccer player

**DOI:** 10.1186/s40634-023-00690-0

**Published:** 2023-11-22

**Authors:** Luis Alberto Buendia Saavedra

**Affiliations:** Hospital San Ángel Inn, Benito Juárez, México City, Mexico

**Keywords:** Achilles tendon rerupture, Full-thickness reconstruction, Clearant Process graft, Bio Composite Arthrex screw, Innovative fixation, Imaging follow-ups, Functional outcomes, Case study, Soccer player

## Abstract

We present a case of a 41-year-old male amateur soccer player with no comorbidities, who experienced a rerupture of the Achilles tendon 5 years after his initial end-to-end plasty. To address this, we opted for a full-thickness reconstruction using a graft under the Clearant Process of the Achilles tendon. As an innovative approach, we proposed an alternative fixation technique, employing a Bio Composite Arthrex 9 mm x 35 mm interference screw, placed at the apex of the calcaneus body. For a period of 5 years, the patient underwent regular imaging follow-ups with Magnetic Resonance Imaging (MRI) and clinical assessments using the Foot and Ankle Ability Measure Activity Subscale Score and Foot and Ankle Ability Measure Sports Subscale Score. This case highlights the importance of exploring novel fixation methods for Achilles tendon reconstruction, particularly in cases of rerupture. The use of the Bio Composite Arthrex screw, in conjunction with the Clearant Process graft, demonstrated promising results both in imaging and functional outcomes, but more case studies with positive results are needed to evaluate the effectiveness of this reconstruction.

## Theoretical framework

Rerupture of the Achilles tendon is a complication that can occur after initial treatment, whether it was managed conservatively or surgically. This is a significant concern, as it can lead to prolonged recovery and functional impairment. Here's an overview of rerupture and its treatment:

### Achilles tendon rerupture incidence

Achilles tendon reruptures are not uncommon and can occur following both surgical and non-surgical treatments. The incidence rate varies across studies but is generally reported to be around 3–5% [1]. Understanding the incidence is essential for patient counseling and follow-up care.

### Fixation methods

Various fixation methods have been explored in the literature, with open surgical repair and percutaneous repair being the most common approaches. Open surgery involves making a larger incision to access the ruptured tendon and repair it, while percutaneous repair is minimally invasive, involving smaller incisions and often using special instruments for fixation. The choice of fixation method may depend on factors such as patient age, activity level, and surgeon preference. Additionally, the type of suture material used can impact outcomes, with studies comparing materials like non-absorbable sutures to absorbable sutures [2].

### Complications and risk factors

Achilles tendon reruptures can lead to various complications, including infections, delayed wound healing, and adhesions. Identifying risk factors for reruptures is crucial for preventing these complications. Studies have examined risk factors such as age, smoking status, and obesity, as well as surgical factors like the type of suture material used and the technique employed during repair [3].

### Rehabilitation protocols

Rehabilitation plays a crucial role in the recovery of Achilles tendon ruptures, whether treated surgically or conservatively. Early mobilization and functional rehabilitation are emphasized in contemporary protocols. Studies like that by Nilsson-Helander et al. [4] have demonstrated that early weight-bearing and range of motion exercises can lead to good functional outcomes and reduced rerupture rates.

### Innovations in fixation methods

Recent innovations in Achilles tendon fixation methods include the use of bioabsorbable anchor systems. These systems can offer the advantage of reducing the risk of implant-related complications and avoiding the need for implant removal surgeries. Grassi et al. [5] investigated the use of a bioabsorbable anchor system for Achilles tendon repair and reported early positive outcomes.

Motivated by the need for advancements in this area, we present our surgical technique, along with its clinical and imaging follow-up, with the aim of inspiring replication and improvement for cases involving significant losses in the Achilles tendon.

## Objective

The aim of this study is to propose a surgical technique for Achilles tendon reconstruction in cases with losses greater than 5 cm up to 10 cm. Our approach involves utilizing a full-thickness Achilles tendon graft processed under the Clearant Process, with intraosseous fixation at the apex of the calcaneus body using a Bio Composite interference screw placed in a retrograde manner.

## Materials and methods

We present this case of a 41-year-old male patient with no comorbidities, who experienced sudden and disabling pain in the left heel while playing soccer. Upon examination in the emergency room, a re-rupture of the Achilles tendon was diagnosed using ultrasound. It is worth noting that the patient had a significant medical history, including a left Achilles tendon rupture 5 years prior, which was treated with end-to-end plasty without any complications. The patient leads an active lifestyle, participating in amateur soccer, regular running, amateur surfing, and office work. As the chosen treatment, Achilles tendon reconstruction was decided, employing an Achilles tendon graft processed under the Clearant Process, with a full-thickness graft.

### Surgical technique

**Fig. 1:**
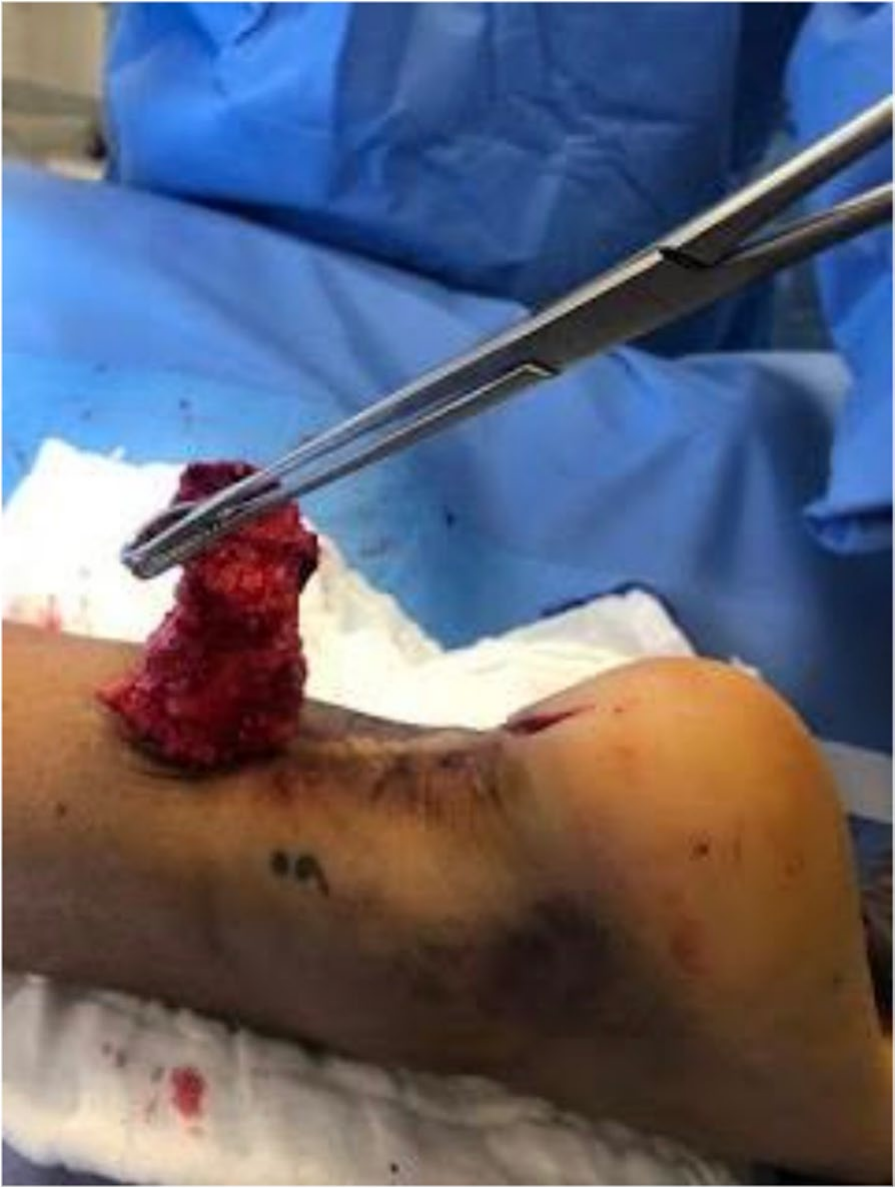
Meticulous 6 cm distal tendon stump debridement﻿

**Fig. 2:**
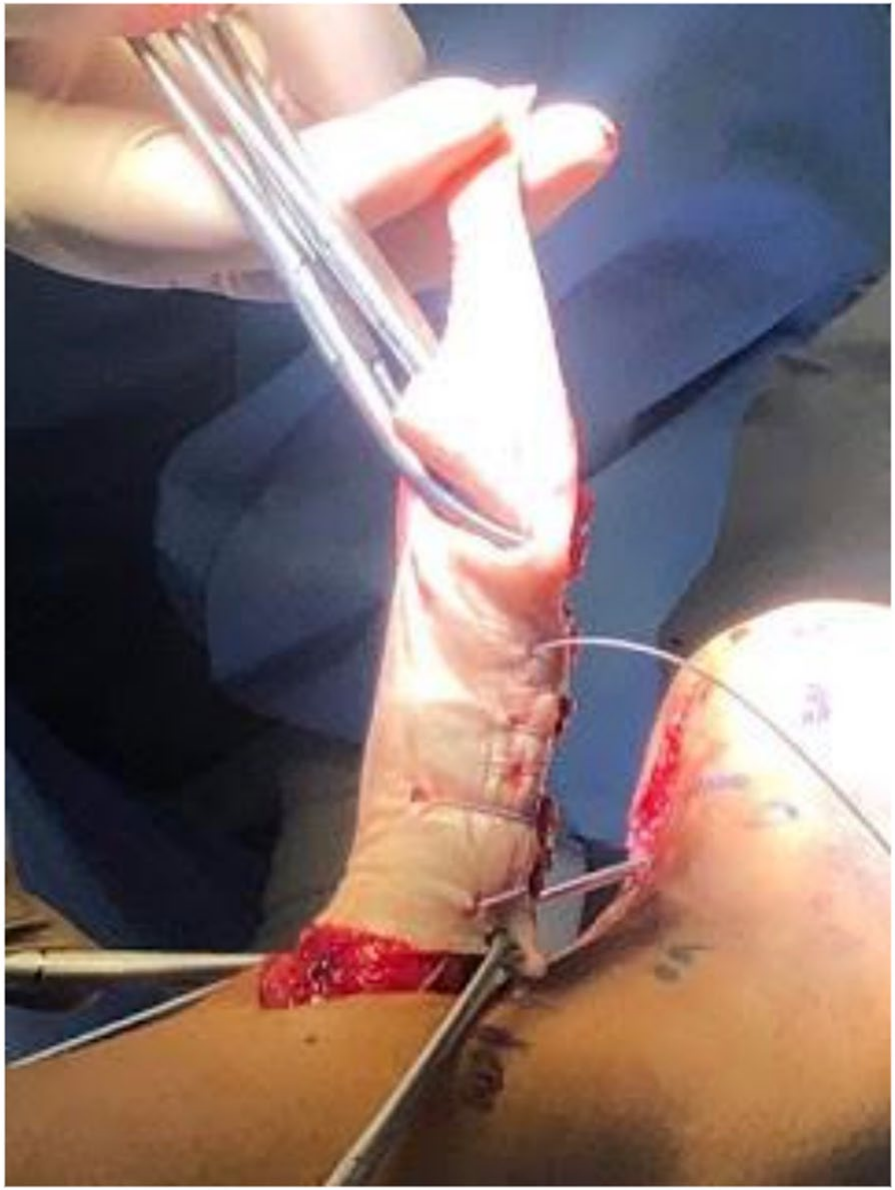
Suturing Graft with Fiberwire 2.0 in Krackow Points

**Fig. 3:**
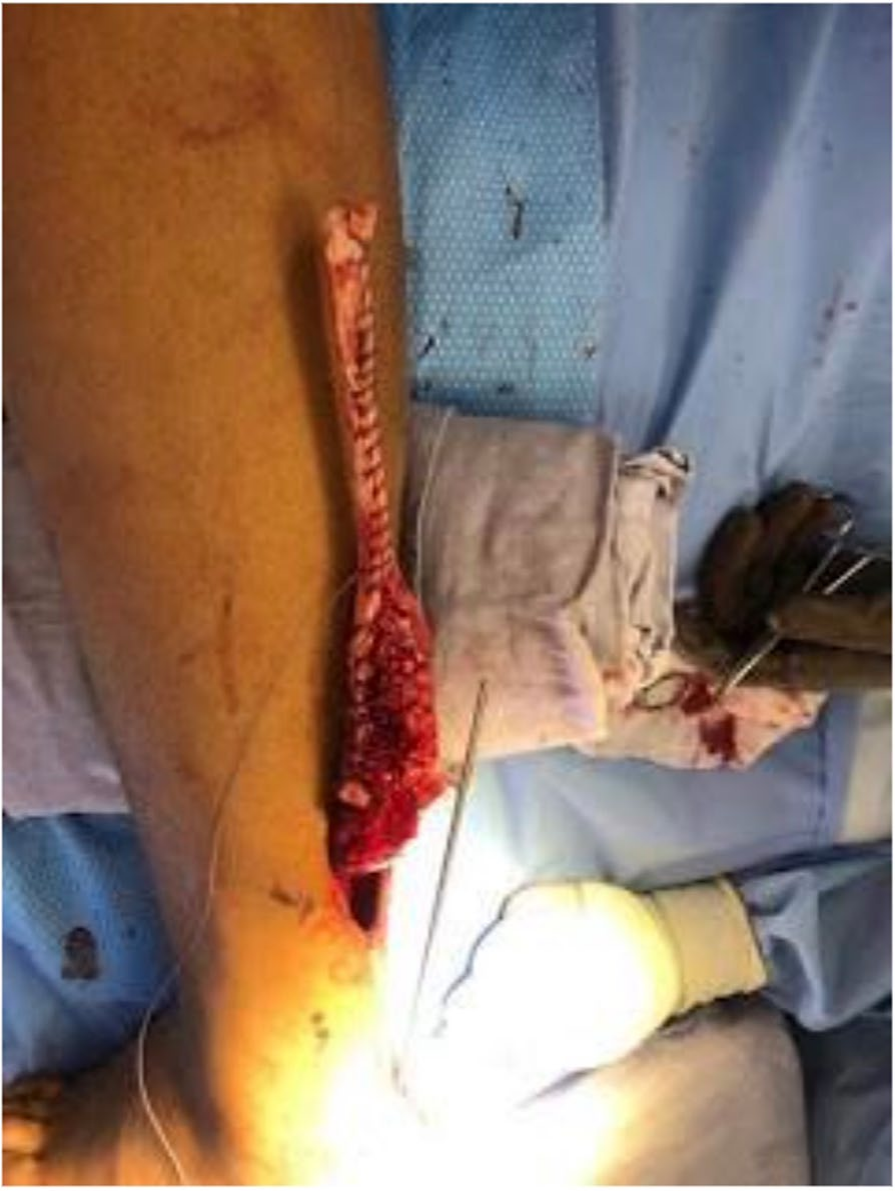
Fluoroscopy-Guided Tunnel Creation in the Calcaneus Apex Using Drill Guide Equipment

**Fig. 4:**
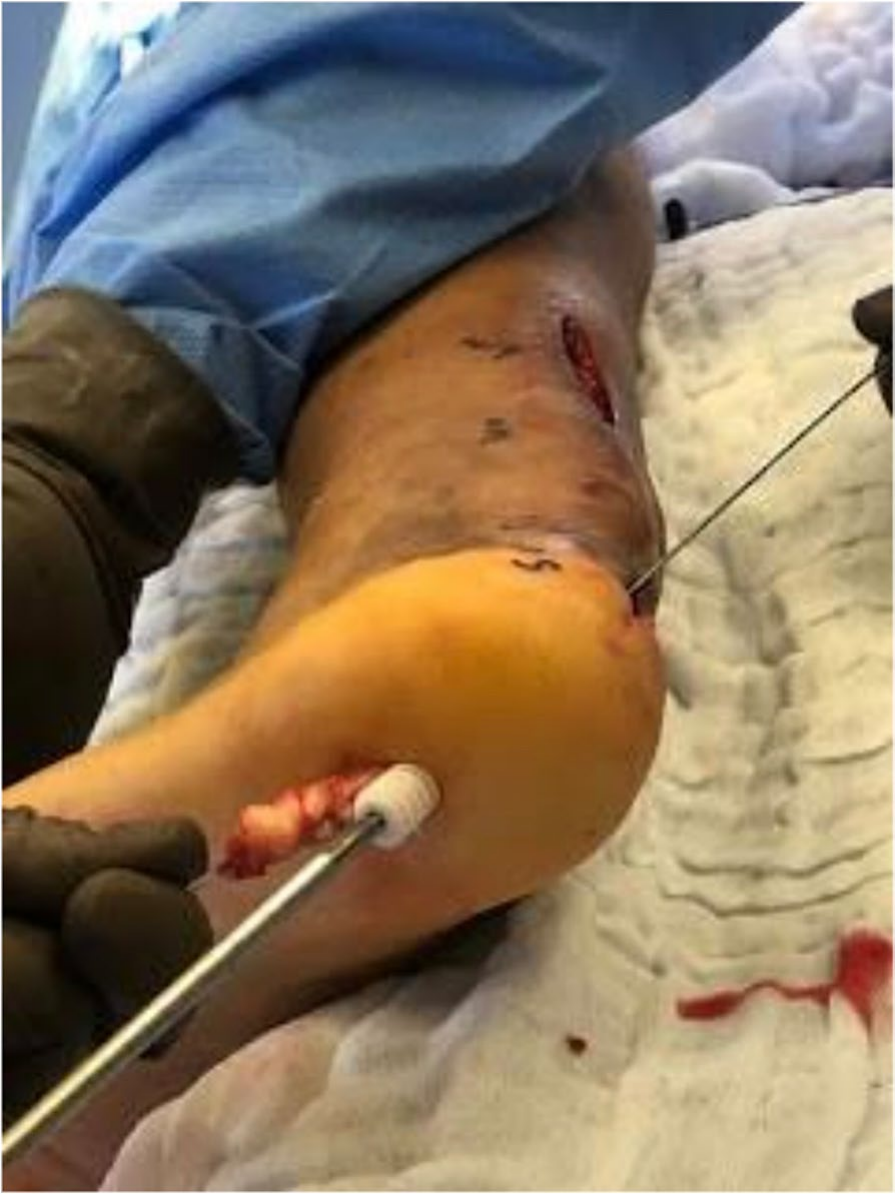
Retrograde Fixation with 9 mm x 35 mm Arthrex Bio Composite Interference Screw

**Fig. 5:**
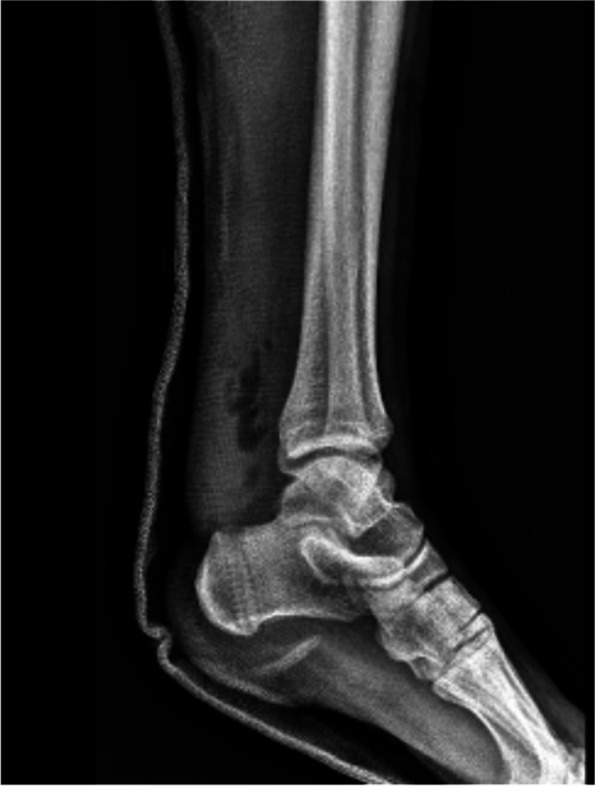
Radiographic control for implant depth assessment in calcaneus anteroposterior and lateral projections

**Fig. 6:**
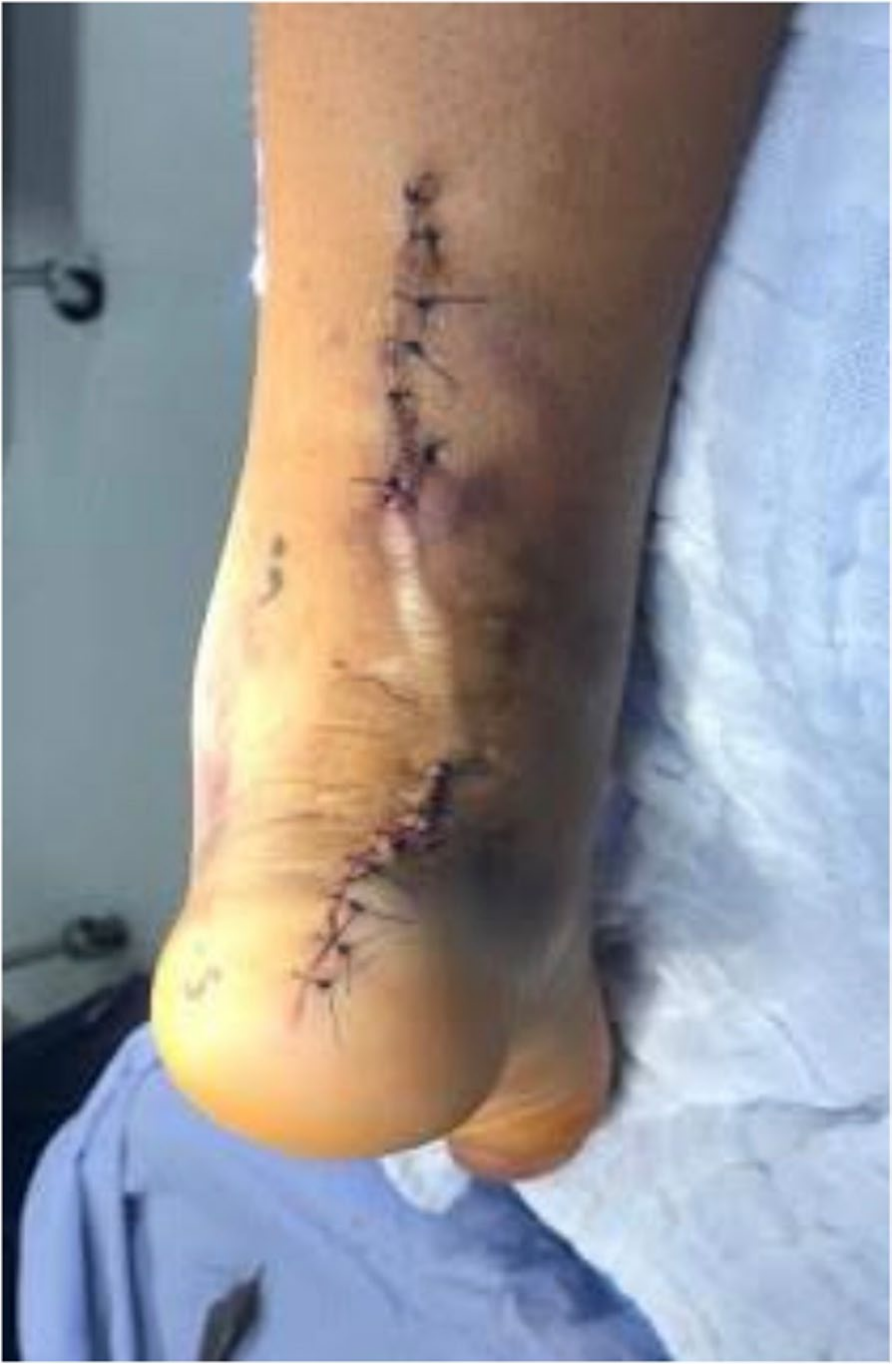
Closure of surgical approaches with meticulous application of Non-absorbable Sutures in the skin

The Achilles tendon reconstruction procedure involved the utilization of a full-thickness graft processed under the Clearant Process. Initially, the graft underwent washing in physiological solution, followed by intratendinous infiltration with 10 cc of autologous PRP without activator. The surgery was performed under regional anesthesia, ventral recumbent position; maintaining asepsis suffice and antisepsis of the thigh and leg with 0.12% chlorhexidine gluconate and pneumatic ischemia to 240 mmHg 45 min. Following the midline of the central posterior approach, two discontinuous approaches of 5 cm each are performed; The first goes from the posterior and superior edge of the calcaneus to 5 cm proximal, the second goes from 15 to 20 cm proximal from the posterior and superior edge of the calcaneus. A 6 cm section of the distal tendon stump was meticulously debrided until non-fibrous tissue was exposed (Fig. 1). The graft was sutured into place using Fiberwire 2.0 in Krackow points (Figs. 2 and 3). Subsequently, the anterior cruciate ligament equipment was employed, beginning with a drill guide and assisted by fluoroscopy, to create a tunnel in the calcaneus at the apex level of the calcaneal body, directed centrally between the medial and lateral processes of the calcaneus. The drilled tunnel was calibrated to match the graft diameter, in this instance, 9 mm. To ensure optimal muscular balance; was checked with repeated flexions and extensions until proper balance was noted prior to fixation with strong traction. (QR 1). For retrograde fixation, a 9 mm × 35 mm Arthrex Bio Composite interference screw was used (Fig. 4). To determine the implant depth, radiographic control was conducted, encompassing anteroposterior (AP) and lateral projections of the calcaneus within the operating room (Fig. 5). The surgical approaches were then meticulously closed with non-absorbable sutures in the skin (Fig. 6). The pneumatic ischemia was discontinued, and the overall surgery duration was 45 min.

### Rehabilitation protocol integration

The presented case study underscores the complexity of managing Achilles tendon reruptures and the necessity of innovative surgical techniques. To complement these surgical advancements and facilitate optimal recovery, we now integrate a comprehensive rehabilitation protocol, synthesized from existing literature.

Phase I: Early Post-Operative (Weeks 0–2)

- Goals: Minimize swelling, control pain, protect the repair, and facilitate wound healing.
- Interventions:
- Consistent with the case, the patient remains in a non-weight-bearing cast or boot, ensuring adequate rest and protection.
- Elevation and cryotherapy to mitigate postoperative swelling and pain.
- Initiation of gentle active range of motion exercises within the pain-free range, under the guidance of a physical therapist.
- Medication for pain management as needed, supervised by the medical team.

Phase II: Intermediate (Weeks 2–6).

- Goals: Gradual restoration of range of motion, improved weight-bearing tolerance, and progressive strengthening.
- Interventions:
- Transition to a controlled ankle motion (CAM) boot (Week 4), allowing early weight-bearing as prescribed.
- Initiation of passive dorsiflexion and plantarflexion exercises, with continued supervision by the physical therapist.
- Introduction of protected weight-bearing progression, monitored closely for patient comfort and compliance.
- Commencement of eccentric calf muscle strengthening exercises under physiotherapist supervision.
- Initiation of balance and proprioception training.

Phase III: Late Post-Operative (Weeks 6–12).

- Goals: Full range of motion, normalized gait pattern, and increased strength.
- Interventions:
- Transition to full weight-bearing, with the CAM boot gradually phased out (Weeks 6–8).
- Continued stretching and strengthening exercises, with a focus on achieving full range of motion and optimal calf muscle strength.
- Gait retraining to promote a normal stride and movement pattern.
- Initiation of low-impact activities (e.g., stationary biking, aquatic therapy) as deemed appropriate by the physical therapist.

Phase IV: Functional and Return to Activity (Months 3–6).

- Goals: Restoration of functional strength and sport-specific activities.
- Interventions:
- Gradual transition to high-intensity loading exercises.
- Introduction of agility and sport-specific drills, emphasizing controlled movements.
- Plyometric training to rebuild explosive strength and improve proprioception.
- Collaboration with a sports therapist to guide the patient's gradual return to sports activities in a structured and monitored manner.

Phase V: Maintenance and Long-Term Management (After 6 Months).

- Goals: Sustained functional recovery, prevention of reinjury, and maintenance of overall lower limb strength.
- Interventions:
- Continued adherence to strength and conditioning exercises.
- Periodic follow-up assessments to monitor progress and address any residual issues.
- Emphasis on injury prevention strategies, including a thorough warm-up routine, appropriate footwear, and biomechanical assessments, as well as ongoing guidance from a physical therapist or athletic trainer.

## Results

**Fig. 7:**
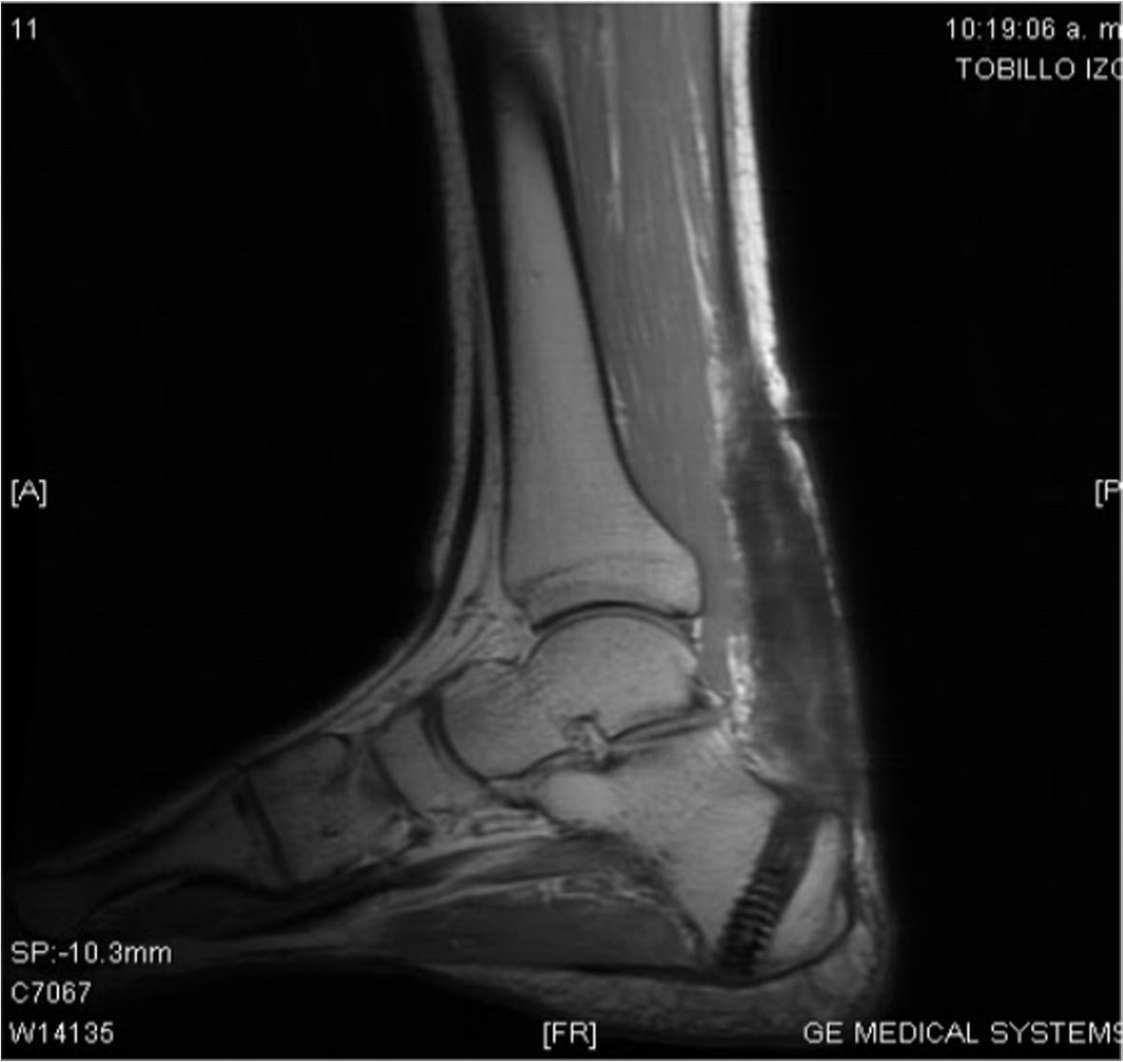
Sagittal plane MRI of left ankle - hypointense achilles tendon with fusiform appearance, enhanced by Karger's fat, hyperintense on T1 and T2 with fat saturation

**Fig. 8:**
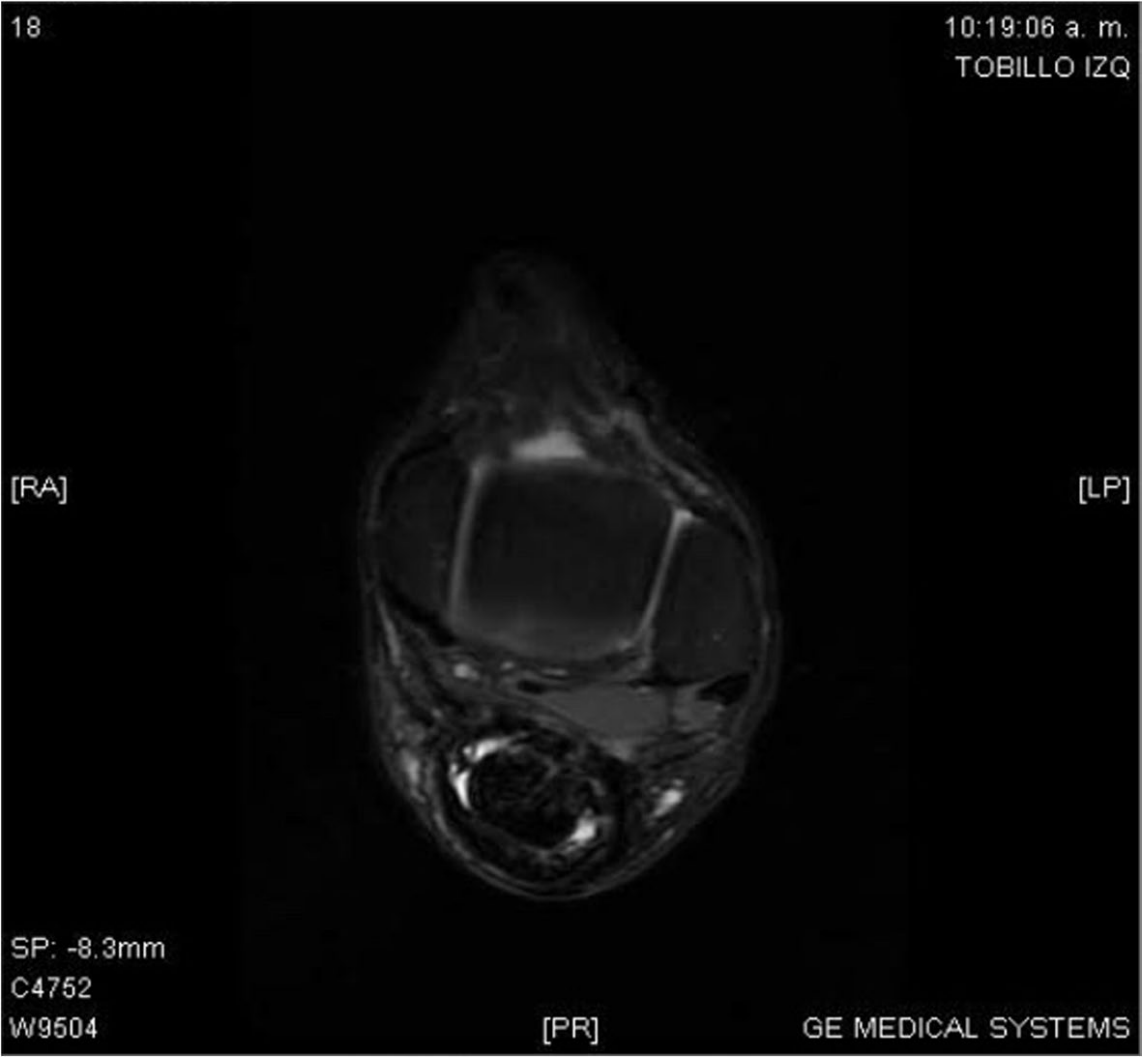
Transverse plane MRI of left ankle - hypointense Achilles tendon with fusiform appearance, enhanced by Karger's fat, hyperintense on T1 and T2 with fat saturation

The patient underwent a regular follow-up schedule, with weekly visits for the first three months, followed by monthly visits. Notably, the clinical progression demonstrated a rapid and progressive recovery of function and gait within seven weeks (QR 2). By twelve weeks post-surgery, there was a gradual resumption of low-impact sports activities, with the graft displaying successful integration and biomechanical functionality. Functional follow-up was conducted monthly during the first year after the surgery and annually thereafter, utilizing the Foot and Ankle Ability Measure (FAAM). At the 5-year mark after the surgery, the Foot and Ankle Ability Measure Activity Subscale Score reached an impressive 84 out of 84 points or 100%, indicating a high level of functional recovery. Similarly, the Foot and Ankle Ability Measure Sports Subscale Score attained 30 out of 32 points or 94% (QR 3), further demonstrating the patient's excellent progress in sports-related activities. These outcomes signify the successful implementation of the proposed surgical technique, leading to a significant improvement in the patient's functional abilities and quality of life over the 5-year follow-up period. MRI follow-up at 11 months after surgery revealed the adequate integration and revascularization of the reconstructed Achilles tendon, reaching more than 90%. The MRI images of the left ankle displayed a hypointense Achilles tendon, clearly delineated by Karger's fat, exhibiting a tendon with a fusiform appearance and appearing more hyperintense on T1 and T2 sequences with fat saturation (Figs. 7 and 8). Cross-sectional MRI of the left ankle showed a mostly homogeneous, hypointense Achilles tendon with a 15 mm caliber on both T1 and T2 sequences. These MRI findings provide further evidence of the successful healing and integration of the reconstructed Achilles tendon, demonstrating its improved vascular supply and structural integrity. The observed changes in signal intensity and morphology indicate a positive outcome and support the favorable functional results observed in the patient's clinical follow-up.

## Discussion

The demographic profile of our patient, a middle-aged male athlete with no comorbidities, aligns with the typical presentation of Achilles tendon rerupture cases reported in the literature [3]. Furthermore, the utilization of a Clearant Process graft for Achilles tendon reconstruction is in harmony with established practices employing grafts in such procedures [6].

Our case introduces an innovative fixation method utilizing a Bio Composite Arthrex interference screw, which is a deviation from conventional fixation techniques. This approach resonates with the exploration of novel fixation methods discussed in contemporary literature [5].

We underscore the importance of rigorous follow-up, employing Magnetic Resonance Imaging (MRI) and validated clinical assessments (Nilsson-Helander et al., [4). Our emphasis on monitoring closely aligns with the literature's focus on rehabilitation protocols and the assessment of functional outcomes.

Postoperative Antibiotics: After surgery, the choice and duration of antibiotic treatment should be based on individual patient factors and the surgeon's assessment. The choice may vary depending on local antibiotic resistance patterns. We use 15-day antibiotic prophylaxis with levofloxacin 500 mg every day.

Postoperative (NSAID): The choice of a non-steroidal anti-inflammatory drug (NSAID) in cases of Achilles tendon injuries, including reruptures, should be made by a healthcare provider based on the patient's individual medical history, allergies, and any potential drug interactions. Celecoxib is a selective cyclooxygenase-2 (COX-2) inhibitor that can be used to manage pain and inflammation in various musculoskeletal conditions, including cases where heterotopic ossifications are a concern (Burd, T. A., et al., [7]). We prescribe celecoxib considering heterotopic ossifications for 15 days 200 mg per day.

The presence of exudate in a wound following Achilles tendon reconstruction with a graft can vary depending on several factors, including the surgical technique, patient-specific factors, and postoperative care. While it is not uncommon for some exudate to occur in the early stages of wound healing, excessive or persistent exudate may raise concerns about infection or other complications. It is important to mention that we observed a serous exudate at the distal site of the approach for 2 weeks with a production of 5 cc per day; that worried us for a moment but spontaneously closed. The study by (Hennessy, M. S., et al., [8) investigates the relationship between wound exudate and wound infection in patients who underwent Achilles tendon surgery. It explores the significance of wound exudate as a potential predictor of infection, emphasizing the importance of monitoring and managing exudate in postoperative care.

In the case of our 41-year-old male amateur soccer player who experienced a rerupture of the Achilles tendon five years after an initial end-to-end plasty, we must carefully consider the timing of his return to sport. This scenario aligns with the broader understanding in the literature that the return to sport after Achilles tendon reconstruction with a full-thickness graft can be variable and depends on several critical factors.

1. Individual Factors: Our patient, being an amateur soccer player with no comorbidities, is in relatively good health, which is a positive factor. However, his age and previous injury history may affect the rehabilitation process (Forsdyke D et al.,[9]).
2. Surgical Technique: The choice to perform a full-thickness reconstruction using the Clearant Process graft, along with the innovative fixation technique involving the Bio Composite Arthrex screw, is an important aspect of this case. Surgical techniques can influence the rehabilitation timeline, and the use of this particular graft and fixation method should be monitored closely (Nilsson-Helander et al., [4]; Beard & Dodd, [10]).
3. Rehabilitation Progress: The patient's regular imaging follow-ups with MRI and clinical assessments using the Foot and Ankle Ability Measure Activity Subscale Score and Foot and Ankle Ability Measure Sports Subscale Score are vital for tracking his rehabilitation progress. This monitoring helps gauge when it's safe for the patient to return to sport and can inform the healthcare team about any potential setbacks (Brumann et al., [11]; Järvinen et al., [12]).
4. General Timeline: Based on the literature and the patient's specific situation, we can anticipate that his return to sport may fall within the general range of 6 to 12 months after surgery. However, given the rerupture scenario and the unique graft and fixation approach used, it's essential to approach this case with individualized care and remain flexible regarding the return timeline (Forsdyke D et al., [9]).

One year post-surgery, the patient successfully resumed full integration into amateur sports activities. Over the last four years, he has maintained an active sports regimen, participating twice a month, showcasing both dedication and resilience. Beyond his sporting pursuits, he has also embraced a consistent running routine, engaging in this activity three times a week. Furthermore, he has enjoyed occasional bouts of surfing.

## Conclusions

In discussing the disadvantages of the proposed surgical technique for Achilles tendon reconstruction, it's important to critically examine potential drawbacks and areas of concern without drawing final conclusions. Here's a discussion highlighting some potential disadvantages:

1. Limited Applicability: While the surgical technique is deemed valuable for cases involving irreparable losses greater than 5 cm to 10 cm, it may not be suitable for patients with smaller injuries or those with less severe Achilles tendon damage. This limitation restricts its widespread applicability.
2. Risk of Complications: Any surgical procedure carries inherent risks, and this technique is no exception. The insertion of the graft through a tunnel to the apex of the calcaneus using calibrated drill bits, while intended to be precise, poses the risk of misalignment or graft damage during surgery, which could lead to complications or suboptimal outcomes.
3. Controversial Use of PRP: The use of platelet-rich plasma (PRP) remains a matter of controversy in Achilles tendon reconstruction. While the technique acknowledges its application, it does not definitively address the concerns and uncertainties surrounding PRP's role in graft integration. This may leave room for doubts among both patients and healthcare professionals.
4. Two Approaches in a Single Location: While the use of two approaches in a single location is praised for reducing morbidity and relieving pressure on critical stress points, it may also increase the complexity of the procedure. This could potentially lead to longer surgery times and a higher risk of complications.
5. Need for Further Validation: The study acknowledges that further studies and long-term follow-up are essential to validate the efficacy and reproducibility of the technique. This indicates that its long-term success and safety are not yet firmly established, which can be a source of uncertainty for both patients and surgeons.
6. Specialized Training: Implementing this surgical technique may require specialized training for surgeons, as it involves a unique approach. This could limit its accessibility to patients in regions where such expertise is scarce.
7. Patient-Specific Considerations: It's important to recognize that not all patients are the same. Factors such as age, overall health, and lifestyle can influence the suitability of this technique for individual patients. A one-size-fits-all approach may not address these unique considerations adequately.
